# Supplementary material for: Correlation between Mild Cognitive Impairment and Sarcopenia: The Prospective Role of Lipids and Basal Metabolic Rate in the Link
Source: Nutrients. 2022 Dec 15;14(24):5321. doi: 10.3390/nu14245321 (PMC9783732; doi:10.3390/nu14245321)
Supplement: Supplementary file 1 [file nutrients-14-05321-s001.zip › nutrients-1959640-supplementary.pdf]

Supplementary Material

Detailed Methods

Sarcopenia assessment

Appendicular lean mass is measured by direct segmental multi-frequency bioelectrical impedance analysis (BIA) (InBody 720 analyser, InBody Co., Ltd., Korea). Skeletal muscle index (SMI) was calculated by appendicular skeletal muscle mass of arms and legs. The cut-off points of SMI for sarcopenia is<7.0kg/m^2 for male and<5.7kg/m^2 for female. Smedley-type handgrip dynamometer (Yagami Co, Tokyo, Japan) was used to detect the grip strength. The definitions of low grip strength are<28kg for male and<18kg for female respectively. 6m timed distance was used to evaluate the usual gait speed of natural walk. The 5-chair stand test (5-CST) asks a participant to stand up from a chair and sit back down five times as quickly as possible. The lower level of physical function is defined as gait speed<1.0m/s or 5-CST≤12s for both genders.

Nontargeted Lipidomics

Lipids preparation 250 μL water was added into each 50 μL of erythrocyte lysates. After 30s vortex, the samples were frozen and thawed with liquid nitrogen for 3 times. Then the samples were sonicated for 10 min in the ice-water bath. Next, 50μL was taken out for protein determination, adjust the homogenate concentration according to the protein results. Add 480μL of extraction liquid (V_{MTBE}: V_{MEOH}=5:1) to the EP tube. After 30 s vortex, the samples were sonicated for 10 min in the ice-water bath. Then the samples were incubated at -40°C for 1h and centrifuged at 3000rpm (RCF=900(×g), R= 8.6cm) for 15 min at 4°C. 350 μL of supernatant was transferred to a fresh tube and dried in a vacuum concentrator at 37°C. The dried samples were reconstituted in 150μL of DCM/MeOH (1:1, v/v) by 30s vortex and sonication on ice for 10min. The constitution was then centrifuged at 13000rpm for 15min at 4°C, and 75μL of supernatant was transferred to a fresh glass vial for LC-MS analysis. The quality control (QC) sample was prepared by mixing 10μL of the supernatants from all samples.

Chromatography and mass spectrometry The injection volume was 4μL for positive ion mode and 6μL for negative ion mode, respectively. The column temperature was 55°C. The auto-sampler temperature was 4°C. The UHPLC-QE-MS/MS was equipped with a dual electrospray ionization probe sourced to both ESI^+ and ESI^-. The MS full scan data was executed in both ionization modes: spray voltage of 5kV in ESI^+ and -4.5kV in ESI^-, capillary temperature 320°C in ESI^+ and 300°C in ESI^-, sheath gas flow rate 30Arb and auxiliary gas flow rate 10Arb, full MS resolution as 70000, MS/MS resolution as 17500. All mass spectra were analyzed at 15, 30, 45 normalized collisional energy (NCE). The acquisition software (Xcalibur 4.0.27, Thermo) continuously analyzed the full scan survey MS data while collecting and triggering the acquisition of MS/MS spectra depending on preselected criteria. Data acquisition was performed in the mode of information-dependent acquisition and dynamic background subtraction.

Data preprocessing and annotation The raw data files were converted to files in mzXML format using the MS convert program from ProteoWizard. Peak detection was first applied to the MS1 data. The CentWave algorithm in XCMS was used for peak detection with the MS/MS spectrum, lipid identification was achieved through a spectral match using the Lipid Blast library.

Table S1. The 63 variables incorporated into the LASSO model

**Demographic characteristics**			
age	sex	education	BMR
**Chronic diseases**			
hypertension	diabetes	dyslipidemia	arthritis
**Serum Cholesterol**			
TC	TAG	HDL-C	LDL-C
**Dietary intakes**			
energy	protein	CHO	fat
cholesterol	total fatty acid	SFA	C4:0
C6:0	C8:0	C10:0	C11:0
C12:0	C13:0	C14:0	C15:0
C16:0, PA	C17:0	C18:0	C19:0
C20:0	C22:0	C24:0	MUFA
C14:1	C15:1	C16:1, POA	C17:1
C18:1, OA	C20:1	C22:1	C24:1
PUFA	C16:2	C18:2, LA	C18:3
C20:2	C20:3	C20:4, AA	C20:5, EPA
C22:3	C22:4	C22:5, DPA	C22:6, DHA
total choline	betaine	free choline	GPC
phosphatidylcholine	PC	SM	

AA, arachidonic acid; BMR, basal metabolic rate; CHO, carbohydrate; DHA, docasahexaenoic acid; DPA, docosapentaenoic acid; EPA, eicosapentaenoic acid; GPC, glycerol phosphatidylcholine; HDL-C, high density lipoprotein cholesterol; LA, linoleic acid; LDL-C, low density lipoprotein cholesterol; MUFA, monounsaturated fatty acid; OA, oleic acid; PA, palmitic acid; PC, lecithin; POA, palmitoleic acid; PUFA, polyunsaturated fatty acid; SFA, saturated fatty acids; SM, sphingomyelin; TC, total cholesterol; TAG, triglyceride.

Table S2. The variables selected by LASSO in each group

	**MCI** vs. **Ctrl**	**SA** vs. **Ctrl**	**MCI&SA** vs. **Ctrl**
**Demographic characteristics**			
	education	gender	gender
	BMR	age	age
		education	education
		BMR	BMR
**Chronic diseases**			
	hypertension	hypertension	hypertension
		dyslipidemia	arthritis
**Serum Cholesterol**			
	TC	HDL-C	LDL-C
	TAG	LDL-C	
**Dietary intakes**			
	betaine	CHO	protein
	phosphatidylcholine	GPC	betaine
	C18:0	C13:0	phosphatidylcholine
	C20:0	C19:0	C19:0
	C24:0	MUFA	C24:0
	C15:1	C20:1	C15:1
	C20:1	PUFA	C22:1
	C24:1	C18:3	C24:1
	C22:4	C20:2	C18:3
	C22:5, DPA	C20:4, AA	C22:5, DPA
		C22:5, DPA	

AA, arachidonic acid; BMR, basal metabolic rate; CHO, carbohydrate; DPA, docosapentaenoic acid; GPC, glycerol phosphatidylcholine; HDL-C, high density lipoprotein cholesterol; LDL-C, low density lipoprotein cholesterol; MUFA, monounsaturated fatty acid; PUFA, polyunsaturated fatty acid; TAG, triglyceride; TC, total cholesterol.

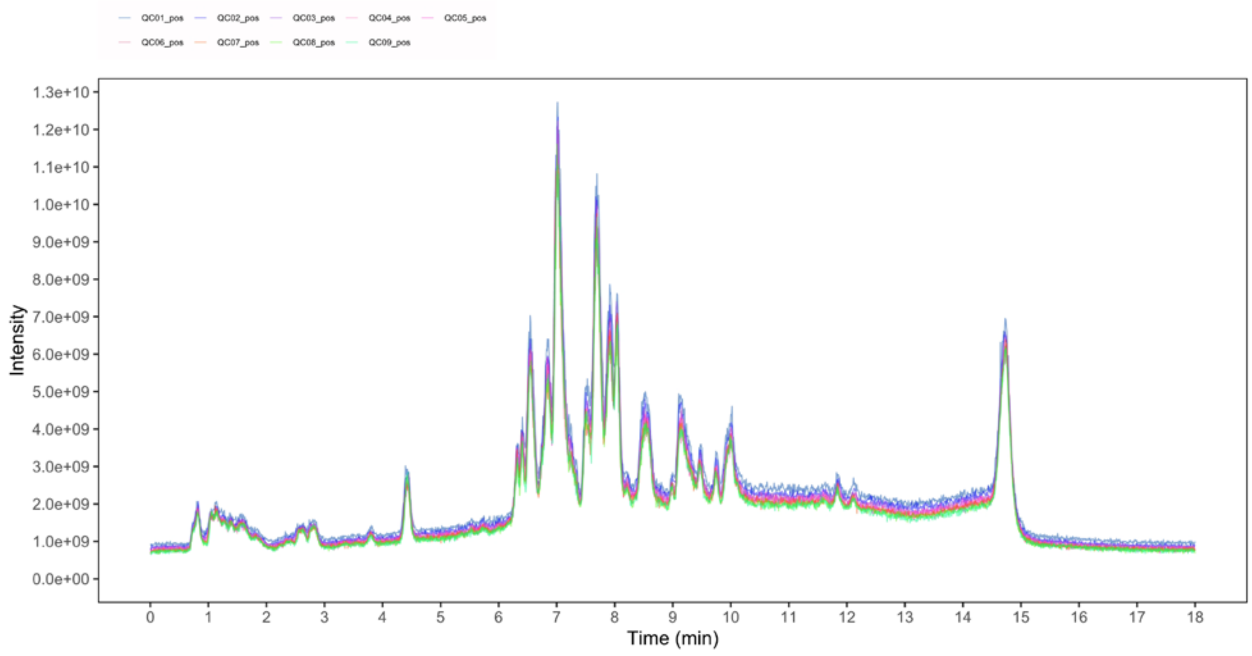

(a)

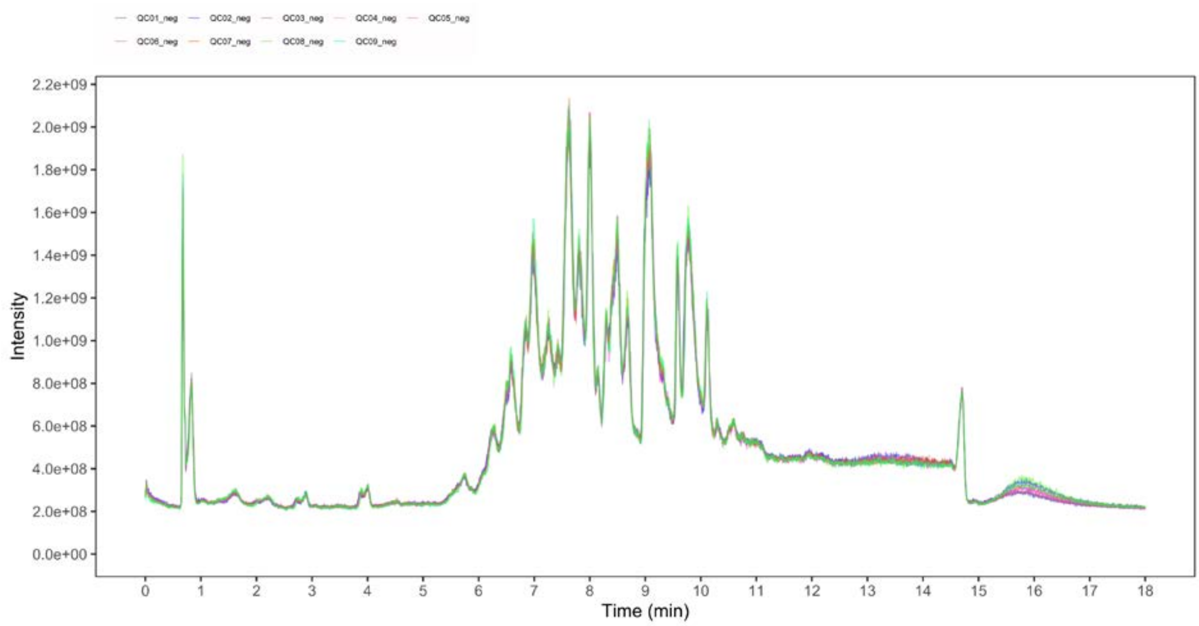

(b)

Figure S1. A typical total ion chromatogram (TIC) of the pooled erythrocytes quality control sample acquired in ESI (+) mode (a) and ESI (−) mode (b) of nontargeted lipidomics.

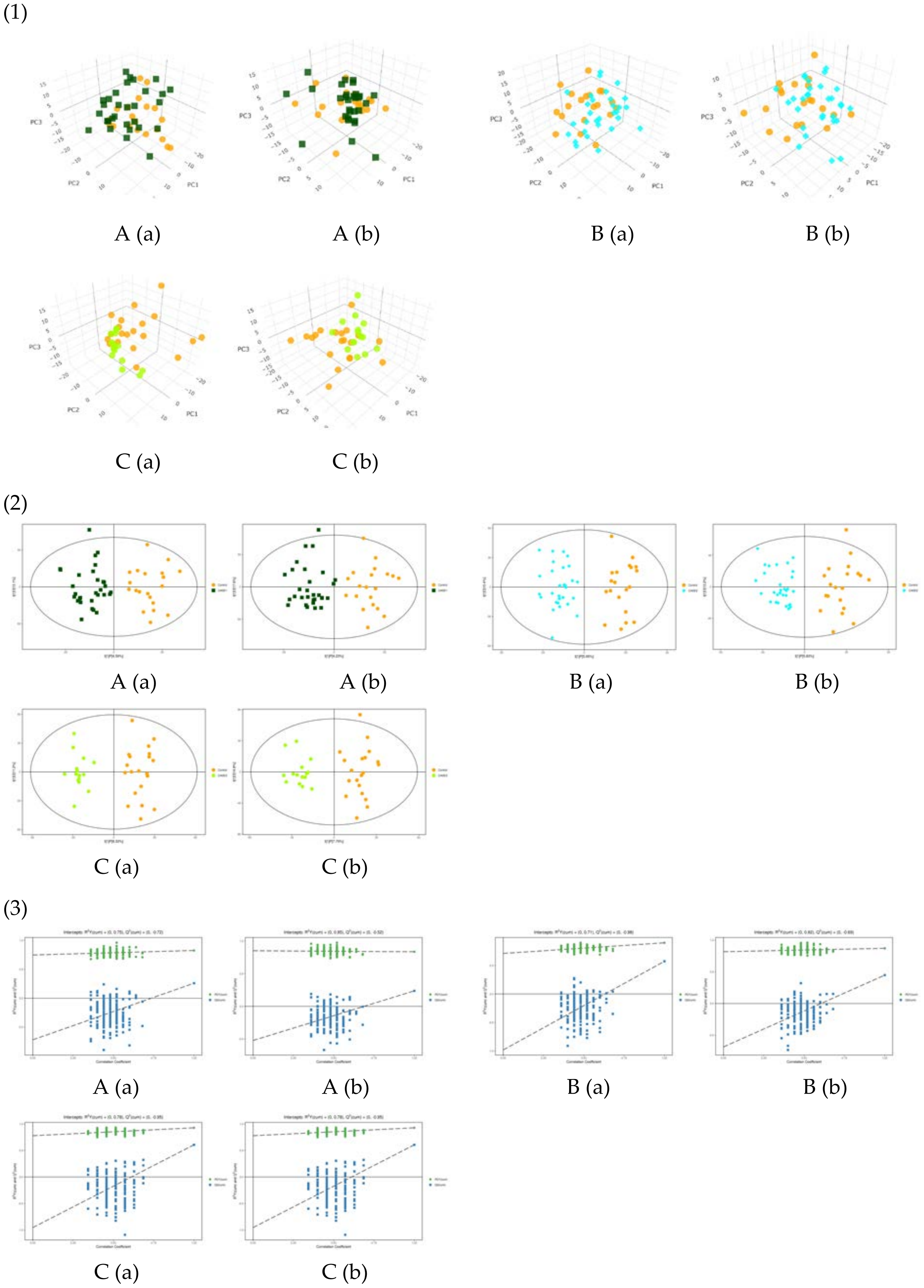

Figure S2. Multivariate analytical workflow with (1) 3D PCA score plot; (2) OPLS-DA score plot; (3) Validation plot of 200 permutation tests for OPLS-DA model in nontargeted lipidomics. (A) MCI vs. Ctrl group; (B) SA vs. Ctrl group; (C) MCI&SA vs. Ctrl group. (a) positive ionization mode; (b) negative ionization mode.

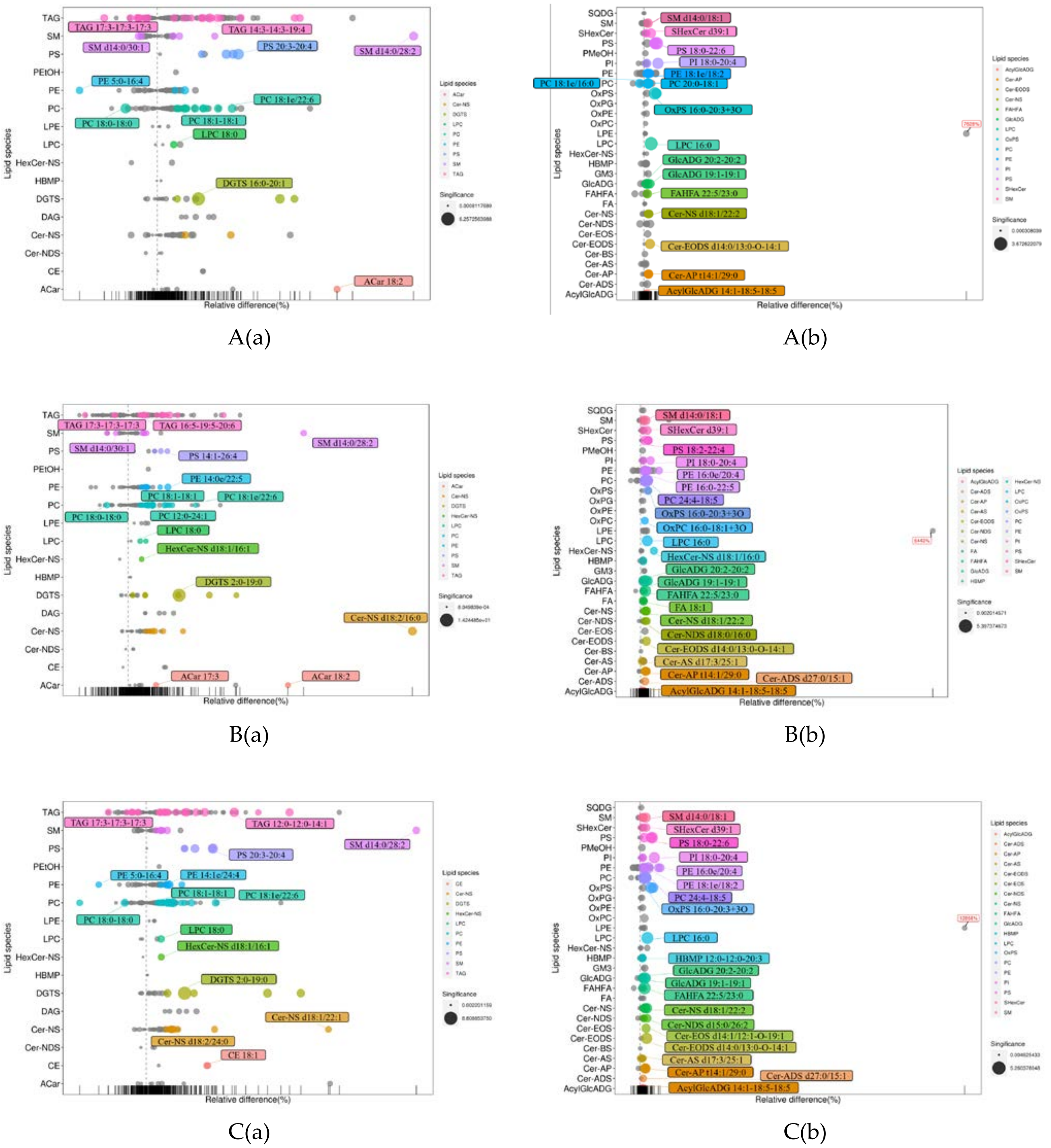

Figure S3. Univariate analytical workflow with bubble plot by UHPLC-QE-MS. (A) MCI vs. Ctrl group; (B) SA vs. Ctrl group; (C) MCI&SA vs. Ctrl group. (a) positive ionization mode; (b) negative ionization mode. CE, cholesteryl ester; Cer, ceramides; DAG, diacylglycerols; DGTS, diacylglyceryltrimethylhomoserine; FFA, free fatty acids; GlcADG, glucuronosyldiacylglycerol; HexCer, hexosylceramide; LPC, lyso-phosphatidylcholines; PC, phosphatidylcholine; PE, phosphatidylethanolamines; PI, phosphatidylinositol; PS, phosphatidylserine; SM, sphingomyelins; TAG, triglycerides.

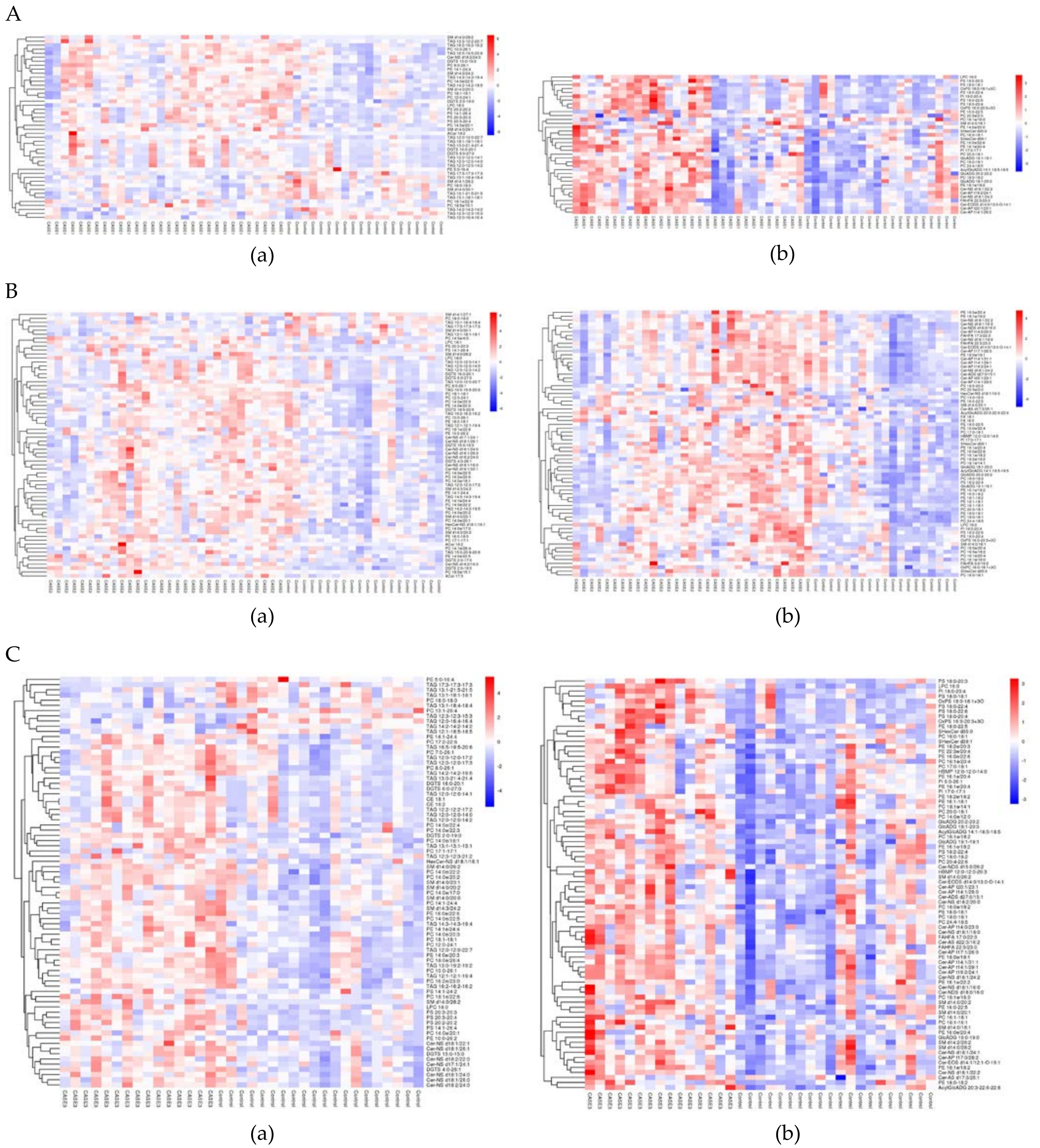

Figure S4. The heat map with hierarchical clustering to determine any erythrocytes lipidome discrepancies in three comparisons between cases and control in nontargeted lipidomics. (A) MCI vs. Ctrl group; (B) SA vs. Ctrl group; (C) MCI&SA vs. Ctrl group. (a) positive ionization mode; (b) negative ionization mode.

Table S3. Sixty key differential lipid species which have the same trend of regulation in three comparisons in nontargeted lipidomics

NO.	Compound (ion mode)	Fold change			*VIP*			*p* value			measured m/z	RT
		MCI vs. Ctrl	SA vs. Ctrl	MCI&SA vs. Ctrl	MCI vs. Ctrl	SA vs. Ctrl	MCI&SA vs. Ctrl	MCI vs. Ctrl	SA vs. Ctrl	MCI&SA vs. Ctrl		
1	AcylGlcADG 14:1-18:5-18:5(-)	1.155	1.156	1.213	1.770	1.556	1.773	0.019	0.015	0.007	987.635	541.351
2	Cer-AP t14:1/29:0(-)	1.246	1.314	1.338	1.545	1.630	1.691	0.028	0.013	0.008	694.628	574.651
3	Cer-AP t19:2/24:1(-)	1.229	1.258	1.370	1.550	1.847	2.148	0.027	0.006	0.001	690.604	545.183
4	Cer-AP t20:1/23:1(-)	1.238	1.289	1.328	1.185	1.505	1.439	0.042	0.013	0.014	692.622	574.789
5	Cer-EODS d14:0/13:0-O-14:1(-)	1.310	1.305	1.450	1.322	1.516	1.861	0.012	0.006	0.000	664.589	544.940
6	Cer-NS d18:1/22:2(-)	1.261	1.315	1.462	1.547	1.932	2.271	0.037	0.006	0.001	662.575	508.619
7	Cer-NS d18:1/24:2(-)	1.216	1.261	1.358	1.428	1.867	2.061	0.034	0.006	0.002	644.598	545.453
8	Cer-NS d18:2/24:0(+)	1.228	1.280	1.322	1.196	1.517	1.656	0.023	0.007	0.004	648.627	568.860
9	DGTS 2:0-19:0(+)	1.335	1.576	1.488	3.071	3.367	2.670	0.000	0.000	0.000	558.436	314.164
10	DGTS 6:0-27:0(+)[*]	2.118	2.224	2.964	2.319	1.767	2.059	0.022	0.015	0.001	726.630	591.192
11	DGTS 15:0-15:0(+)	1.163	1.193	1.269	1.126	1.616	1.922	0.039	0.008	0.002	684.567	539.450
12	DGTS 16:0-20:1(+)[*]	2.000	1.919	2.542	2.664	1.804	2.340	0.001	0.002	0.001	766.669	590.589
13	FAHFA 22:5/23:0(-)	1.171	1.253	1.308	1.409	2.164	2.242	0.026	0.000	0.000	681.580	545.779
14	GlcADG 18:1-20:3(-)	1.104	1.168	1.167	1.861	2.263	1.896	0.025	0.000	0.003	819.554	419.320
15	GlcADG 19:1-19:1(-)	1.310	1.395	1.424	1.429	1.774	1.611	0.026	0.002	0.030	827.629	458.143
16	GlcADG 20:2-20:2(-)	1.176	1.252	1.306	1.920	2.157	1.696	0.010	0.000	0.005	823.568	525.093
17	LPC 16:0(-)	1.369	1.354	1.496	2.935	2.540	2.483	0.000	0.000	0.000	540.331	95.484
18	LPC 18:0(+)	1.135	1.135	1.188	1.485	1.520	1.676	0.035	0.022	0.008	524.371	134.510
19	OxPS 16:0-20:3+3O(-)	1.561	1.381	1.740	2.289	1.357	2.253	0.001	0.012	0.000	832.511	374.542
20	PC 8:0-26:1(+)	1.161	1.207	1.215	1.432	1.737	1.430	0.027	0.003	0.011	760.585	461.715
21	PC 10:0-26:1(+)	1.238	1.275	1.311	1.342	1.670	1.759	0.015	0.001	0.001	788.614	512.811
22	PC 12:0-24:1(+)	1.223	1.276	1.344	1.528	1.852	1.931	0.009	0.000	0.000	788.607	474.870
23	PC 14:0e/20:1(+)	1.496	1.431	1.575	1.451	1.786	1.657	0.007	0.011	0.012	746.596	465.852
24	PC 14:0e/22:5(+)	1.225	1.268	1.336	1.067	1.271	1.350	0.046	0.011	0.009	766.573	437.920
25	PC 16:0-18:1(-)	1.142	1.219	1.225	1.487	1.976	1.524	0.034	0.001	0.005	804.576	490.804
26	PC 18:0-18:1(-)	1.202	1.265	1.305	1.929	2.275	2.199	0.011	0.000	0.000	832.607	534.395
27	PC 18:0-18:2(-)	1.153	1.227	1.264	1.603	2.104	1.996	0.034	0.000	0.001	830.595	471.043
28	PC 18:1-18:1(+)	1.173	1.228	1.271	1.325	1.722	1.813	0.030	0.001	0.002	786.600	475.078

29	PC 18:1e/22:6(+)	1.610	1.498	1.882	2.232	1.097	1.789	0.006	0.018	0.004	818.588	264.280
30	PC 20:0-18:1(-)	1.230	1.265	1.325	1.615	1.286	1.583	0.021	0.010	0.005	860.686	540.592
31	PC 24:4-18:5(-)	1.213	1.305	1.334	2.060	2.531	2.402	0.005	0.000	0.000	900.595	534.034
32	PE 14:1-24:4(+)	1.147	1.211	1.257	1.176	1.350	1.384	0.045	0.007	0.005	766.536	425.545
33	PE 16:0-22:5(-)	1.137	1.228	1.223	2.060	2.531	2.402	0.005	0.000	0.000	900.595	534.034
34	PE 16:0e/22:6(-)	1.184	1.223	1.288	1.358	1.618	1.643	0.023	0.005	0.002	748.529	452.882
35	PE 18:1e/18:2(-)	1.307	1.362	1.555	1.788	1.692	2.293	0.032	0.005	0.000	726.616	514.744
36	PI 17:0-17:1(-)	1.140	1.139	1.174	1.146	1.283	1.442	0.029	0.019	0.014	835.522	480.447
37	PI 18:0-20:4(-)	1.723	1.525	1.966	2.102	2.102	2.266	0.003	0.003	0.000	885.554	352.369
38	PS 14:1-26:4(+)	1.618	1.443	1.839	1.985	1.306	2.168	0.004	0.027	0.001	838.556	383.005
39	PS 18:0-20:3(-)	1.545	1.415	1.793	2.181	1.252	2.118	0.004	0.028	0.004	812.547	401.356
40	PS 18:0-20:4(-)	1.603	1.413	1.776	2.202	1.470	2.140	0.001	0.010	0.001	810.529	374.195
41	PS 18:0-22:6(-)	1.675	1.407	1.816	2.073	1.323	2.122	0.001	0.010	0.000	834.531	364.602
42	PS 20:3-20:3(+)	1.564	1.300	1.619	2.046	1.049	1.965	0.002	0.048	0.001	836.540	381.433
43	SHexCer d35:0(-)	1.117	1.138	1.162	1.679	1.635	1.444	0.041	0.019	0.018	794.548	459.051
44	SHexCer d39:1(-)	1.372	1.361	1.416	1.121	1.361	1.297	0.017	0.013	0.016	848.577	516.480
45	SM d14:0/18:1(-)	1.205	1.276	1.391	1.475	1.226	1.962	0.019	0.003	0.002	719.537	334.024
46	SM d14:0/20:0(+)	1.129	1.178	1.184	1.593	1.600	1.567	0.024	0.004	0.007	705.578	392.844
47	SM d14:0/28:2(+)*	3.089	2.985	4.432	1.816	1.018	1.845	0.004	0.004	0.006	813.681	595.027
48	SM d14:3/24:2(+)	1.180	1.207	1.266	1.337	1.365	1.531	0.033	0.015	0.008	751.544	448.731
49	TAG 12:0-12:0-14:0(+)*	2.162	2.082	2.820	2.420	1.387	1.722	0.019	0.027	0.002	689.574	638.769
50	TAG 12:0-12:0-14:1(+)	1.678	1.561	2.118	2.362	1.496	2.224	0.011	0.023	0.000	687.567	638.608
51	TAG 12:0-12:0-14:2(+)	1.489	1.441	1.743	2.241	1.395	1.964	0.018	0.021	0.001	685.550	607.987
52	TAG 12:0-12:0-22:7(+)	1.369	1.472	1.486	1.730	1.860	1.651	0.011	0.001	0.007	787.594	426.936
53	TAG 14:2-14:2-19:5(+)	1.222	1.215	1.266	1.011	1.444	1.481	0.032	0.013	0.009	797.621	504.276
54	TAG 14:3-14:3-19:4(+)	1.426	1.386	1.513	1.703	1.625	1.616	0.002	0.002	0.001	795.606	490.464
55	TAG 16:2-16:2-16:2(+)	1.360	1.383	1.378	1.275	1.212	1.362	0.020	0.008	0.013	817.648	560.659
56	TAG 16:5-19:5-20:6(+)	1.137	1.177	1.177	1.389	1.803	1.599	0.019	0.001	0.006	895.572	514.811
57	PC 18:0-18:0(+)	0.741	0.760	0.727	2.250	1.804	1.927	0.001	0.004	0.003	790.686	571.878
58	TAG 13:1-18:1-18:1(+)	0.848	0.844	0.817	2.222	1.742	2.362	0.017	0.012	1.734	837.680	597.011
59	TAG 13:1-18:4-18:4(+)	0.837	0.853	0.816	1.847	1.392	1.594	0.014	0.034	0.015	825.621	550.873
60	TAG 17:3-17:3-17:3(+)	0.568	0.513	0.519	1.770	1.766	1.454	0.020	0.010	0.013	853.701	626.543

VIP, Variable Importance in the Projection.

All differential lipid species were selected according to significance evaluation and *VIP* values.

* Fold change almost >2.

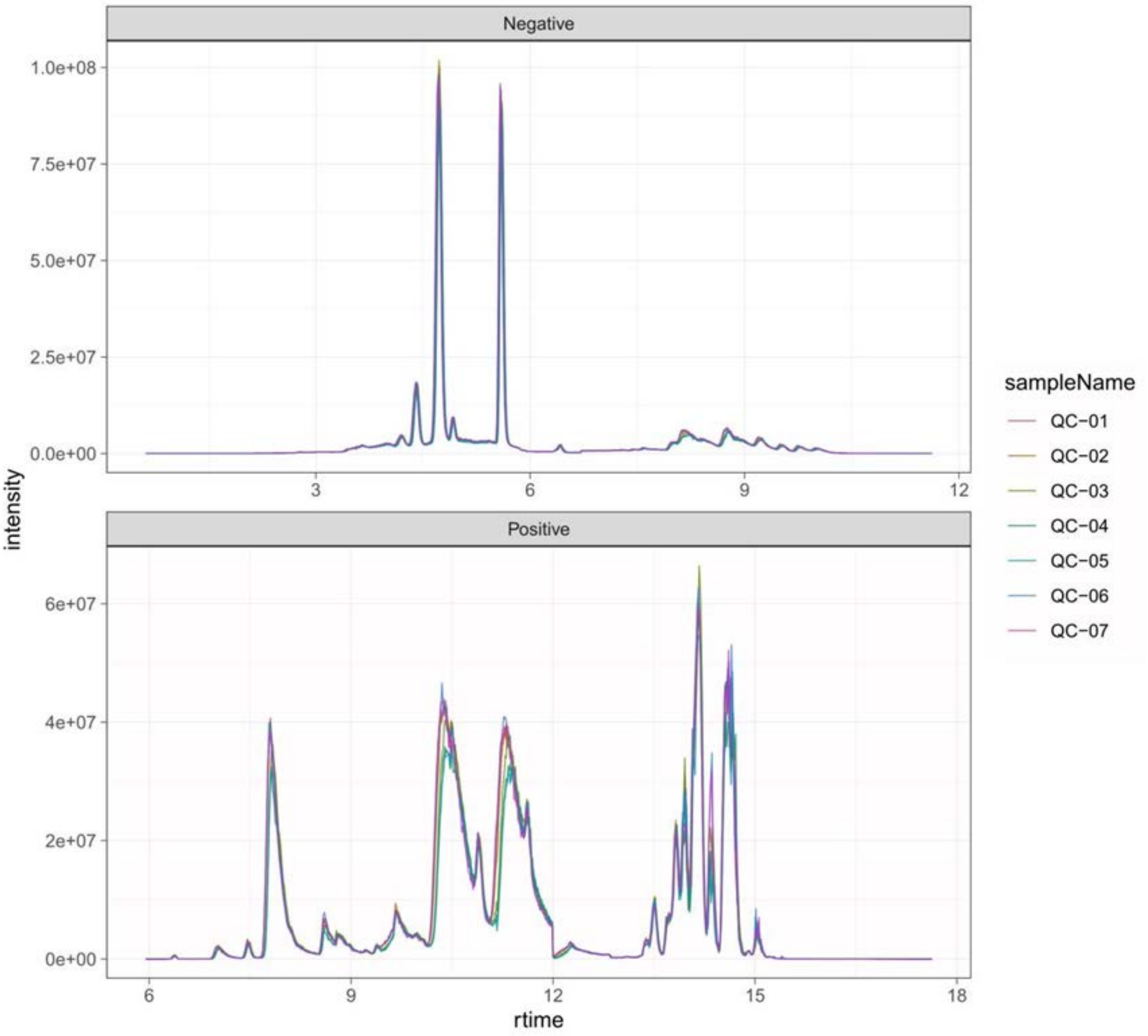

Figure S5. A typical total ion chromatogram (TIC) of the pooled erythrocytes quality control of targeted lipidomics.

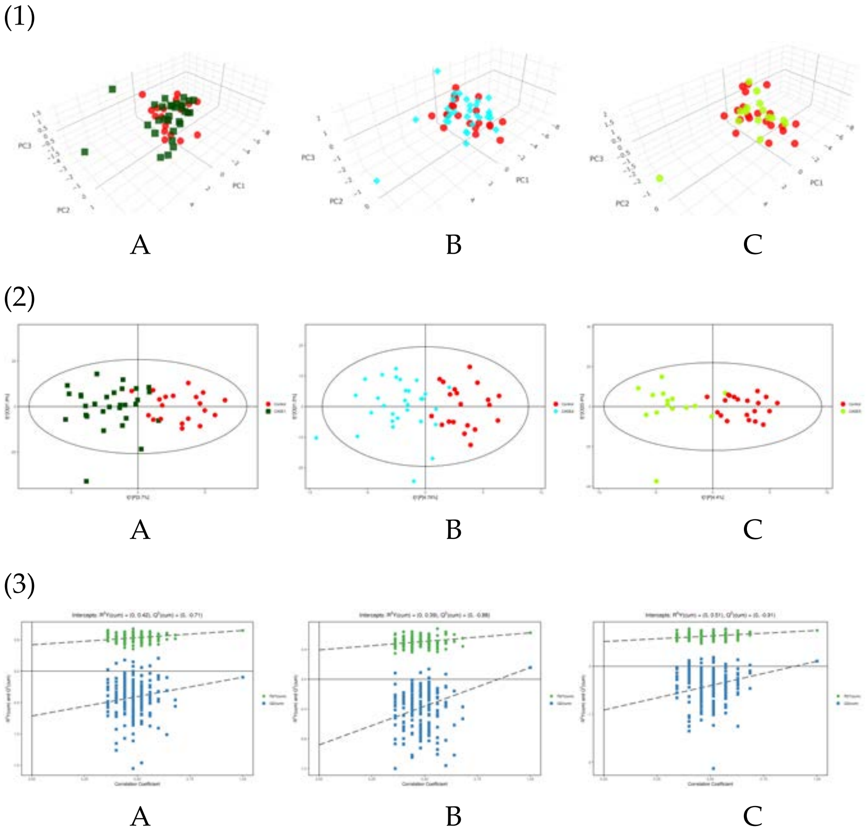

Figure S6. Multivariate analytical workflow with (1) PCA score plot; (2) OPLS-DA score plot; (3) Validation plot of 200 permutation tests for OPLS-DA model in targeted lipidomics. (A) MCI vs. Ctrl group; (B) SA vs. Ctrl group; (C) MCI&SA vs. Ctrl group.

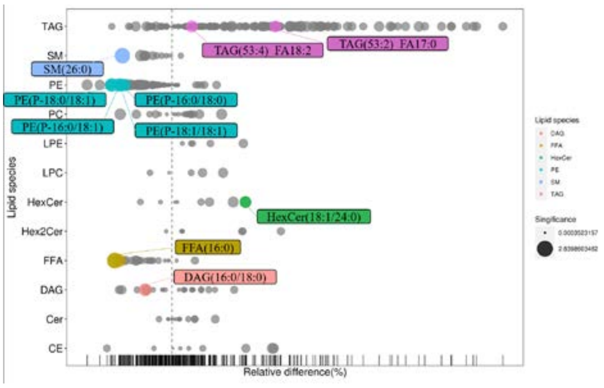

(A)

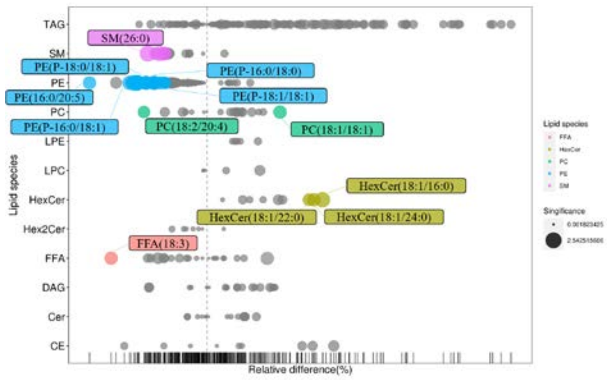

(B)

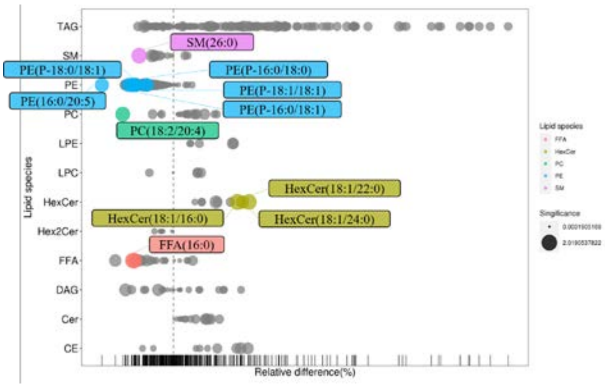

(C)

Figure S7. Univariate analytical workflow with bubble plot by UHPLC-Qtrap-MS. (A) MCI vs. Ctrl group; (B) SA vs. Ctrl group; (C) MCI&SA vs. Ctrl group. (a) positive ionization mode; (b) negative ionization mode. CE, cholesteryl ester; Cer, ceramides; DAG, diacylglycerols; DGTS, diacylglyceryltrimethylhomoserine; FFA, free fatty acids; GlcADG, glucuronosyldiacylglycerol; HexCer, hexosylceramide; LPC, lyso-phosphatidylcholines; PC, phosphatidylcholine; PE, phosphatidylethanolamines; PI, phosphatidylinositol; PS, phosphatidylserine; SM, sphingomyelins; TAG, triglycerides.

(A)

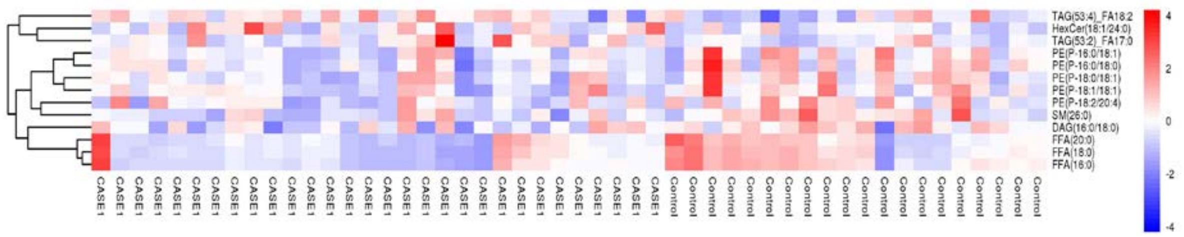

(B)

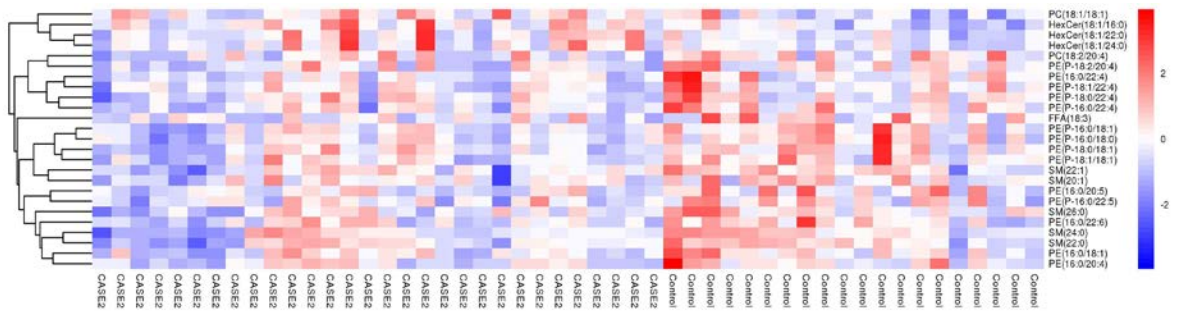

(C)

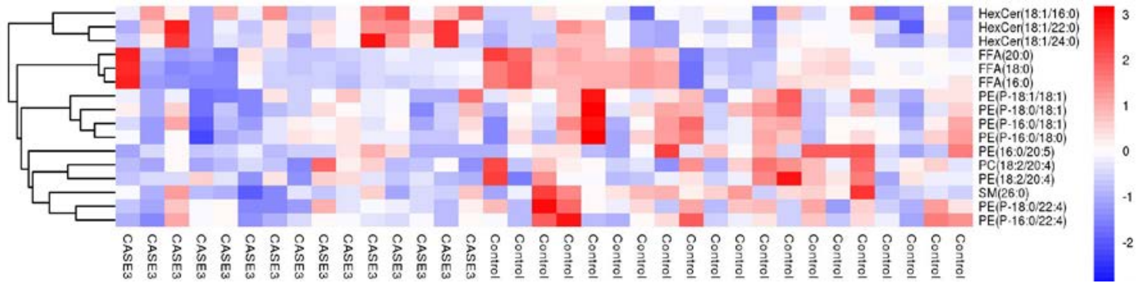

Figure S8. The heat map with hierarchical clustering to determine any erythrocytes lipidome discrepancies in three comparisons between cases and control in targeted lipidomics. (A) MCI vs. Ctrl group; (B) SA vs. Ctrl group; (C) MCI&SA vs. Ctrl group.

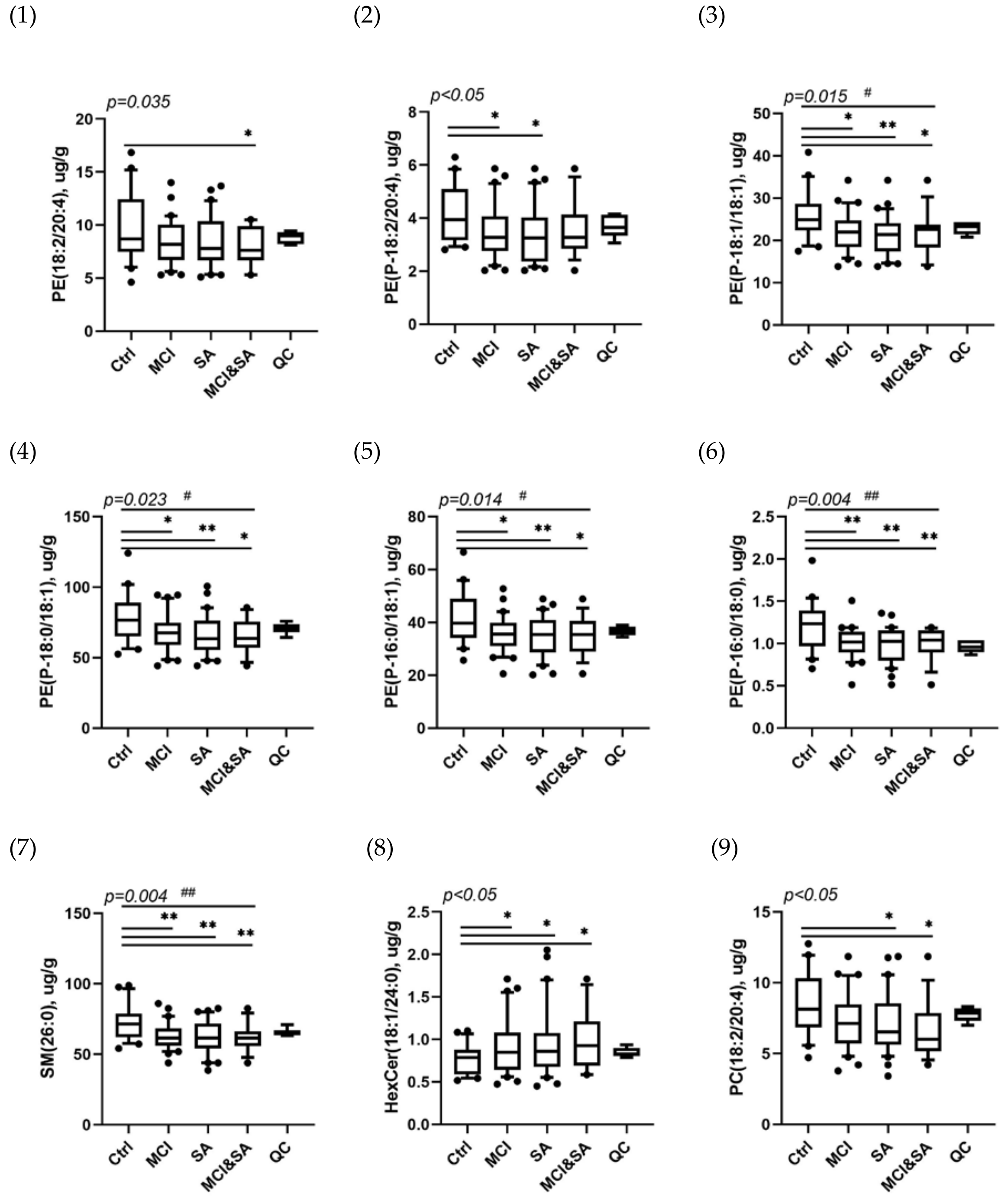

Figure S9. The comparisons of quantitative lipids in profile of erythrocytes based on targeted lipidomic analysis among control group (n=20), MCI (n =30), SA (n=30) and MCI&SA (n=15) groups. Data were shown as Median 10-90 percentile. HexCer, hexosylceramide; PC, phosphatidylcholine; PE, phosphatidylethanolamine; QC, quality control; SM, sphingomyelin. * The differences between case and control group; # The differences among all groups. $^{*}p < 0.05$, $^{**}p < 0.01$; $^{\#}p < 0.05$, $^{\#\#}p < 0.01$.

(1)

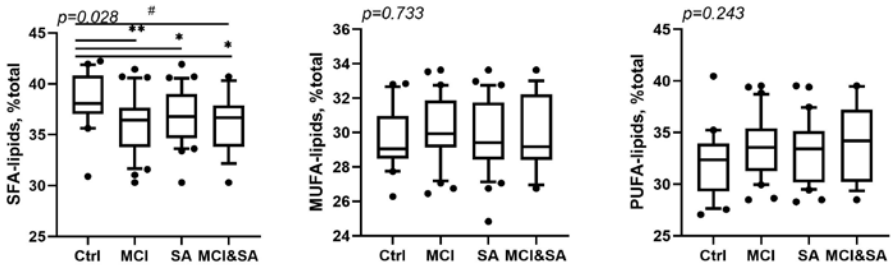
p=0.028
SFA-lipids, %total
p=0.733
MUFA-lipids, %total
p=0.243
PUFA-lipids, %total
Ctrl MCI SA MCI&SA

(2)

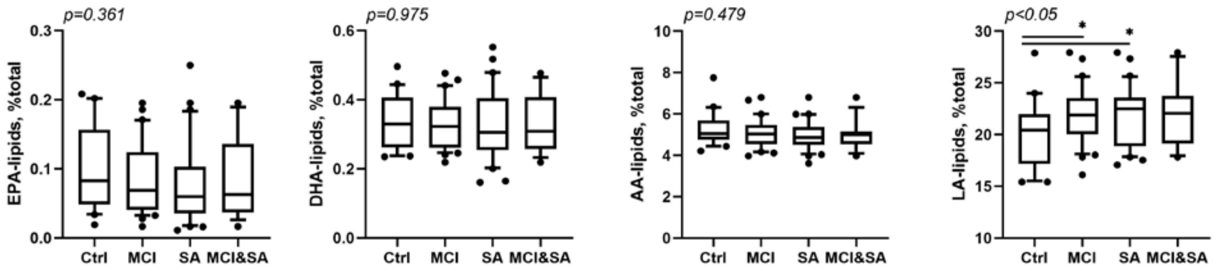
p=0.361
EPA-lipids, %total
p=0.975
DHA-lipids, %total
p=0.479
AA-lipids, %total
p<0.05
LA-lipids, %total
Ctrl MCI SA MCI&SA

(3)

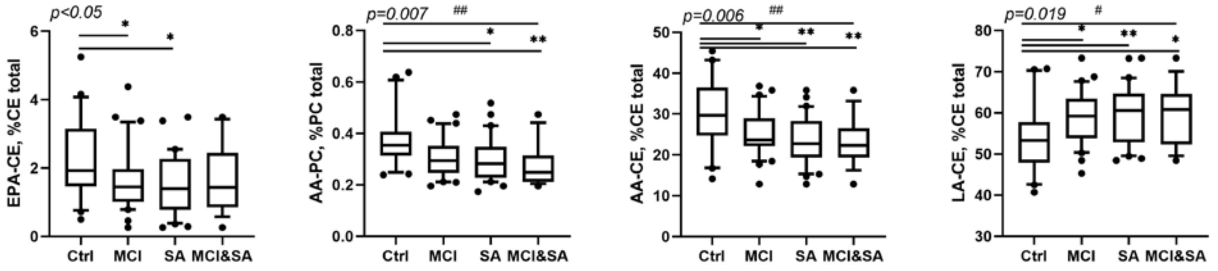
p<0.05
EPA-CE, %CE total
p=0.007
AA-PC, %PC total
p=0.006
AA-CE, %CE total
p=0.019
LA-CE, %CE total
Ctrl MCI SA MCI&SA

(4)

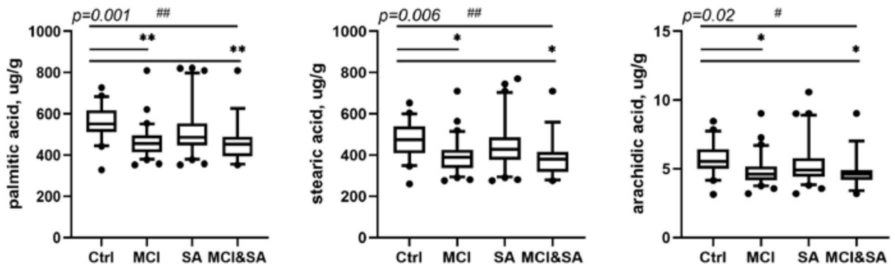
p=0.001
palmitic acid, ug/g
p=0.006
stearic acid, ug/g
p=0.02
arachidic acid, ug/g
Ctrl MCI SA MCI&SA

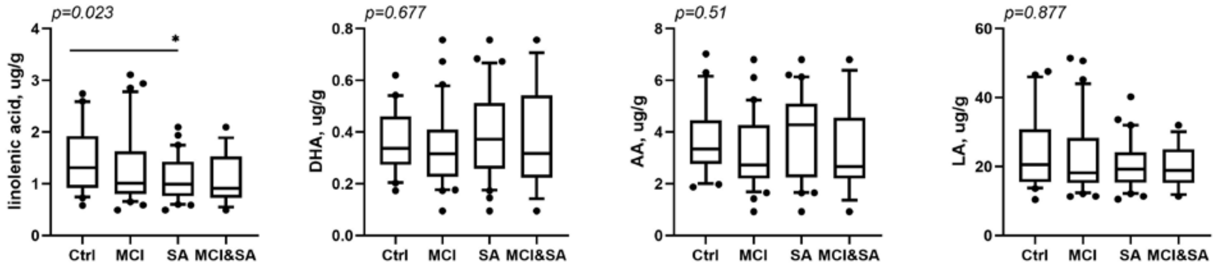
p=0.023
linolenic acid, ug/g
p=0.677
DHA, ug/g
p=0.51
AA, ug/g
p=0.877
LA, ug/g
Ctrl MCI SA MCI&SA

(5)

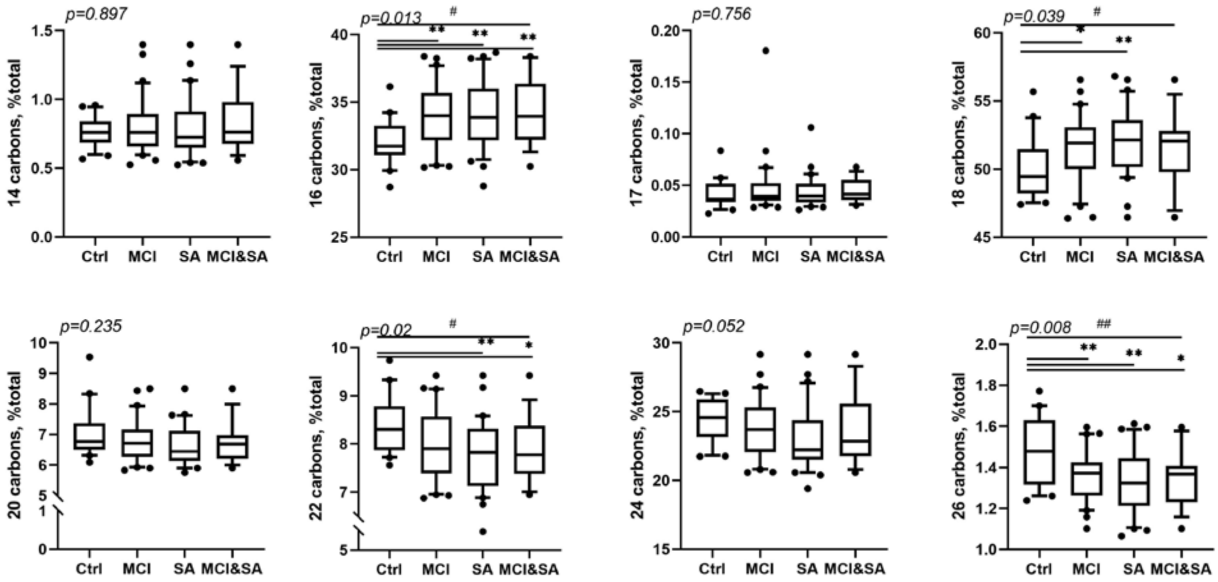

Figure S10. The comparisons of different characteristic lipids in percentage or concentration among different groups. (1) The percentage of lipids containing SFA, MUFA or PUFA acyl chain in total lipids. (2) The percentage of lipids containing EPA, DHA, AA or LA chain in total lipids. (3) The percentage of subclasses of lipid containing EPA, DHA, AA or LA chain, which had statistical difference among groups. (4) The concentration of differential FFAs. (5) The percentage of lipids with acyl chain length over 14 carbons. Data were shown as Median 10-90 percentile. AA, arachidonic acid; CE, cholesteryl ester; DHA, docosahexaenoic acid; EPA, eicosapentaenoic acid; LA, linoleic acid; MUFA, monounsaturated fatty acid; PC, phosphatidylcholine; PUFA, polyunsaturated fatty acid; SFA, saturated fatty acid. * The differences between case and control group; # The differences among all groups. $^{*}p < 0.05$, $^{**}p < 0.01$; $^{\#}p < 0.05$, $^{\#\#}p < 0.01$.
